# A Generic Pixel Pitch Calibration Method for Fundus Camera via Automated ROI Extraction

**DOI:** 10.3390/s22218565

**Published:** 2022-11-07

**Authors:** Tengfei Long, Yi Xu, Haidong Zou, Lina Lu, Tianyi Yuan, Zhou Dong, Jiqun Dong, Xin Ke, Saiguang Ling, Yingyan Ma

**Affiliations:** 1 Aerospace Information Research Institute, Chinese Academy of Sciences, Beijing 100094, China; 2 Department of Eye Disease Control and Prevention, Shanghai Eye Disease Prevention and Treatment Center, Shanghai Eye Hospital, Shanghai 200040, China; 3 Department of Ophthalmology, Shanghai General Hospital, School of Medicine, Shanghai Jiao Tong University, Shanghai 200240, China; 4 Evision Technology (Beijing) Co., Ltd., Beijing 100070, China

**Keywords:** pixel pitch, calibration, fundus image, field-of-view (FOV), optic disc

## Abstract

Pixel pitch calibration is an essential step to make the fundus structures in the fundus image quantitatively measurable, which is important for the diagnosis and treatment of many diseases, e.g., diabetes, arteriosclerosis, hereditary optic atrophy, etc. The conventional calibration approaches require the specific parameters of the fundus camera or several specially shot images of the chess board, but these are generally not accessible, and the calibration results cannot be generalized to other cameras. Based on automated ROI (region of interest) and optic disc detection, the diameter ratio of ROI and optic disc (ROI–disc ratio) is quantitatively analyzed for a large number of fundus images. With the prior knowledge of the average diameter of an optic disc in fundus, the pixel pitch can be statistically estimated from a large number of fundus images captured by a specific camera without the availability of chess board images or detailed specifics of the fundus camera. Furthermore, for fundus cameras of FOV (fixed field-of-view), the pixel pitch of a fundus image of 45° FOV can be directly estimated according to the automatically measured diameter of ROI in the pixel. The average ROI–disc ratio is approximately constant, i.e., 6.404 ± 0.619 in the pixel, according to 40,600 fundus images, captured by different cameras, of 45° FOV. In consequence, the pixel pitch of a fundus image of 45° FOV can be directly estimated according to the automatically measured diameter of ROI in the pixel, and results show the pixel pitches of Canon CR2, Topcon NW400, Zeiss Visucam 200, and Newvision RetiCam 3100 cameras are 6.825 ± 0.666 μm, 6.625 ± 0.647 μm, 5.793 ± 0.565 μm, and 5.884 ± 0.574 μm, respectively. Compared with the manually measured pixel pitches, based on the method of ISO 10940:2009, i.e., 6.897 μm, 6.807 μm, 5.693 μm, and 6.050 μm, respectively, the bias of the proposed method is less than 5%. Since our method doesn’t require chess board images or detailed specifics, the fundus structures on the fundus image can be measured accurately, according to the pixel pitch obtained by this method, without knowing the type and parameters of the camera.

## 1. Introduction

Fundus is a general term for the posterior tissues in the eye, which is the window of human health and the only part of the human body where nerves and blood vessels can be seen noninvasively [1]. The morphological changes in retinal structures such, as the optic disc and blood vessels, are not only related to the occurrence and development of ocular diseases but they are also associated with a variety of systemic diseases. For example, changes in the diameter of retinal vessels are not only considered to be associated with ophthalmic diseases, such as diabetic retinopathy [2] and glaucoma [3], but are also closely related to systemic diseases such as hypertension, arteriosclerosis [4], and chronic kidney diseases [5]. Meanwhile, the size of the optic disc is also reported to be related to ophthalmic diseases, such as acute elevated intraocular pressure [6] and hereditary optic atrophy [7], and systemic diseases, such as multiple sclerosis [8] and Parkinson’s disease [9]. Therefore, fundus photographing has become an irreplaceable tool to obtain the morphological characteristics of retinal structure for the diagnosis and treatment of various diseases, and accurate measurement of fundus structure is also of great importance [10,11].

With the advent of the era of big data, the exploration of the pathogenesis of diseases and evaluation of intervention effects through massive fundus images have become popular forms of research [1]. However, the quantitative measurements of retinal structure, obtained from fundus images taken by different cameras, are often inconsistent due to the variety of camera specifics, which brings trouble to the research, especially for the multi-center design. Therefore, learning how to ensure the consistency of retinal structure measured by different cameras, so that the retinal structural parameters obtained from fundus images can be compared, is of great application value for the study of the pathological mechanism of diseases and the evaluation of intervention effects [12,13].

Although the parameters of the camera can be measured by absolute geometric calibration [14,15], and the pharmaceutical industry standard YYT 0634-2008 and ISO 10940:2009 also provide a method for measuring the pixel pitches of fundus cameras, these methods need to use the uncalibrated camera to take calibration images to estimate the cameras specifics, and the results cannot be applied to other fundus cameras. As the calibration images of the corresponding fundus cameras for the fundus images used in a clinic are usually unavailable, it is difficult to perform traditional geometric calibration for the images. Actually, according to the pharmaceutical industry standards (YYT 0634-2008 and ISO 10940:2009), the designing and settings of fundus cameras need to meet specific requirements, and these constraints make the optic disc an important reference object in fundus images.

The optic disc is one of the important structures of the fundus. In view of its single characteristics, it is often used as a reference to measure the size of other structures or lesions of the fundus in many clinical studies. Therefore, the study of optic disc segmentation and morphological parameters has always been a hot spot in the clinical research of medical imaging, and a variety of disc segmentation approaches were proposed by employing different computer vision algorithms, e.g., adaptive morphology [16], saliency maps [17], multi-layer Mumford Shah vector model [18], super-pixel and SVM classifier [19], combination of morphology, ellipse fitting, and gradient vector flow (GVF) snake model [20], as well as visual attention mechanism [21]. With the development of deep learning, many researchers [22,23,24,25,26,27] have also applied deep learning to the study of disc segmentation and achieved good results. However, most of these studies are based on public data sets, and the computer-aided segmentation approaches were seldom applied to the studies of optic disc morphological parameters, which are important for quantitative assessment in both clinical and public health research. Table 1 summarizes the sizes of the optic discs of population reported by the research literature. Most of these results were obtained based on a single data source and manual measurement, the involved data is usually limited. As the shape of the optic disc is not a regular circle, subjective deviation is inevitable in manual measurement, and research on multi-source and large sample data has great limitations. However, it can be seen from Table 1 that the results of existing studies on the average optic disc size are relatively consistent. Some studies provided the horizontal diameter and vertical diameter, while others only reported the area of the optic disc or the average diameter in two directions. This paper will make use of the maximum diameter of the optic disc area to avoid the influence of the irregular shape; thus, the vertical diameter, which is greater than the horizontal diameter, is considered. Table 2 shows the results of the Meta-analysis of vertical diameter in Comprehensive Meta-Analysis software (CMA) with a random-effects model, and it can be seen that the average value of the vertical diameter is 1.921 ± 0.026 mm.

In this manuscript, we propose a generic and automated pixel pitch calibration approach for fundus cameras based on the geometric modeling of fundus cameras and the analysis of more than 40,000 fundus images. Without the availability of the fundus camera or the knowledge of detailed specifics of the fundus camera, the proposed method can statistically estimate the average pixel pitch, i.e., the actual size of a pixel in the fundus, according to the automatically measured diameter of ROI (region of interest) in a pixel. Comparisons of the pixel pitches measured by the present method and the traditional method based on the method of ISO 10940:2009 were conducted. After obtaining the pixel pitch, our method can be used to measure the size of the optic disc and the diameter of the retinal blood vessels in addition to accurately determining the size of the lesion in the fundus image. While the diameters of the retinal blood vessels are thought to be associated with incidence of cardiovascular and cerebrovascular diseases, the size of the optic disc is thought to be related to neurological disorders. Making the structures in fundus images quantitatively quantifiable and the fundus images acquired by various cameras quantitatively comparable will also aid in measuring the lesion of retinal diseases, such as macular degeneration and diabetic retinopathy, as well as monitoring the progression of the diseases.

## 2. Materials and Methods

### 2.1. Dataset Description

The database used in this study is made up of fundus examination data of community residents in Shanghai, including non-mydriatic fundus images with 45° field-of-view (FOV), age, gender, refraction, etc. The study follows the declaration of Helsinki, and each subject signed a written informed consent form and was approved by the ethics committee of the Shanghai First People’s Hospital Affiliated to Shanghai Jiao Tong University. The age of the subjects ranged from 18 to 96, with an average age of 66 (std = 8), of which 44.5% are male, and all fundus images were automatically evaluated by the quality evaluation system of fundus images and manually verified to ensure the quality based on the clarity and exposure of fundus image. Then, we classified the fundus images according to the fundus cameras, and finally, 40,600 fundus images are used to carry out the research of this paper, including 9600 fundus images taken by Topcon NW400 (Topcon, Tokyo, Japan), 10,000 images taken by Canon CR2 (Canon, Tokyo, Japan), 11,000 images taken by Zeiss Visucam (Carl Zeiss, Jena, Germany), as well as 200 and 10,000 images taken by Newvision Reticam 3100 (New Vision, Chongqing, China).

### 2.2. Methodology

#### 2.2.1. Overall Technical Route

Instead of manually measuring the pixel pitch of the fundus image (the actual size, in fundus, of a pixel in the fundus image) according to ISO 10940:2009, this work will try to directly estimate the pixel pitch from the fundus images without the accessibility of the fundus camera. However, as the size of a human optic disc individually varies significantly, it is not possible to figure out the magnification of the fundus image according to the optic disc size in the image alone. Fortunately, the size of the imaging area for a specific fundus camera of fixed FOV will be much more stable. In consequence, the region of interest (ROI), which is defined as the area where the fundus structure is visible in the fundus image, is applied, in this paper, to measure the constant area of the image. The ROI is, generally, a circular area located in the center of the fundus image, and it can be detected and measured automatically. Once the actual size of ROI in fundus is known, the average pixel pitch of the fundus image can be directly estimated by dividing the diameter of ROI in fundus by the diameter in the pixel.

As the average sizes of human optic discs reported by different studies are basically consistent with each other (the standard deviation is less than 0.1 mm, according to Table 2), the diameter of the optic disc can, generally, be obtained with a high confidence as long as a large number of samples are considered. Although the pixel pitch cannot be determined from a single image without knowing the detailed specifics of the camera, it can be estimated according to the size of ROI, which is constant and related to the average diameter of the optic disc. For instance, the diameter of the ROI in fundus is constant for 45° fundus cameras, and the ratio of the diameter of ROI and the mean diameter of the optic disc is stable according to the following theoretical analysis.

(1) Theoretical basis

**Figure 1:**
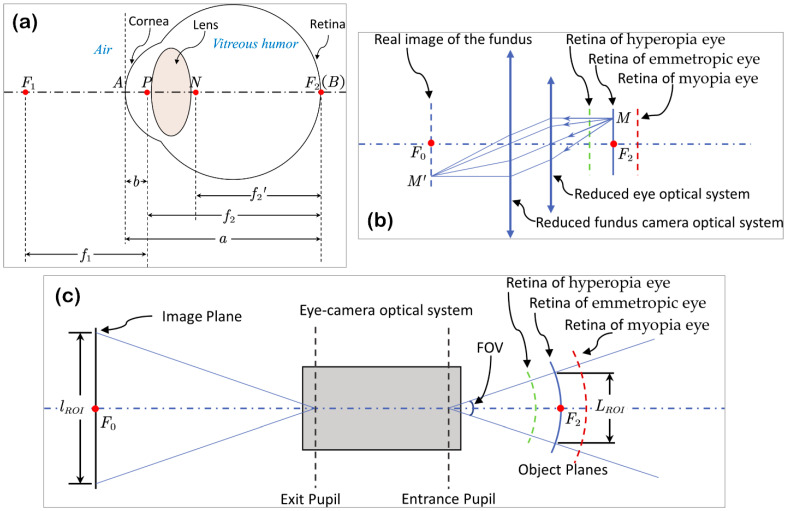
Geometric bases of the eye and the fundus camera model. (**a**) shows the reduced emmetropic eye model, (**b**) shows simplified optical process of fundus camera, and (**c**) shows the geometric model of the FOV of a fundus camera.

The human eye can be considered as a compound optical system consisting of a corneal portion and a lens portion. The former portion refracts the rays by separating air from aqueous humor, while the latter portion subsequently refracts the rays by separating aqueous from vitreous humor, and the rays finally travel to the retina in vitreous humor. Briefly, the reduced model eye, a simplified version of the Gullstrand schematic eye [40], is illustrated in Figure 1a. In this model, the eye is considered as a single refractive surface optical system, and there are four cardinal points along the eye axes, i.e., two focal points (*F*_1_ and *F*_2_), the principal point (*P*), and the nodal point (*N*) [41]. As the nodal point keeps the direction of the incident rays, it is remarkably useful to calculate the magnification of the optical system.

The axial length of the eye is the distance from the corneal surface to the retinal pigment epithelium, and for an emmetropic eye, it is the distance (*a*) from the anterior pole (*A*) to the second focal point (*F*_2_), which is coincident with the retina (*B*). The first focal length (*f*_1_) of the eye is the distance between the first focal point (*F*_1_) and the principal point (*P*), while the second focal length in vitreous humor (*f*_2_) is the distance between the principal point (*P*) and the second focal point (*F*_2_). Generally, the refractive index of air is 1, while that of the vitreous humor is 1.336; for an emmetropic eye, *f*_1_ = *f_2_’* ≈ 17 mm, *a* ≈ 23.87 mm.

As in the reduced emmetropic eye model, the retina is coincident with the second focal plane of the eye optical system, the fundus camera performs like a telescope together with the eye, and the optical rays between the eye and the camera will form a parallel beam. The imaging process of the fundus camera can be described by Figure 1b. Note that the retina layer near the eye axis is illustrated as a plane for simplification.

For a point *M* in the retina, its corresponding point *M’* in the fundus image will appear in the focal plane of the camera, and the magnification of the fundus image, i.e., the ratio of $\bar{M^{\prime }F_{0}}$ and $\bar{MF_{2}}$, will equal the ratio of $f_{0}$ (the focal length of fundus camera) and $f_{1}$ (the focal length of the emmetropic eye in the air). For a myopia eye or hyperopia eye, the retina layer locates behind or in front of the second focal plane of the eye and the fundus camera needs to be refocused, and the magnification of the fundus image can be approximately calculated by

(1) $$m=\frac{f_{0}}{\left(f_{1}+\Delta d\right)}$$

where Δd is the positive or negative distance between the retina layer and the second focal plane of the eye. Δd can be calculated from Formula (2) if the axial ametropia of the eye is provided as D.

(2) $$\Delta d=\frac{-f_{1}^{2}D}{1+f_{1}D}$$

According to Formula (2), a change of 1 mm in axial length can approximately produce a refractive error of three diopters, as well as result in 5.9% bias in fundus image magnification compared with that of the emmetropic eye. Otherwise, the fundus image magnification can be considered as constant if the images are captured by the same fundus camera. The geometric process of capturing a fundus image with a specific field of view can be illustrated as shown in Figure 1c. Here, the retina is considered as a spherical surface, as a plane can no longer accurately approximate the retina for the full FOV.

The diameter of the fundus image ROI in fundus can be calculated from the FOV and the radius of the retina sphere,

(3) $$L_{ROI}=\alpha \left(f_{1}+\Delta d\right)$$

where α is the FOV of the fundus camera, and $f_{1}+\Delta d$ approximates the radius of the retina sphere.

The average pixel pitch of the fundus image is

(4) $$\bar{P}=\frac{L_{ROI}}{l_{ROI}}$$

where $l_{ROI}$ is the length of ROI diameter in pixels.

**Figure 2:**
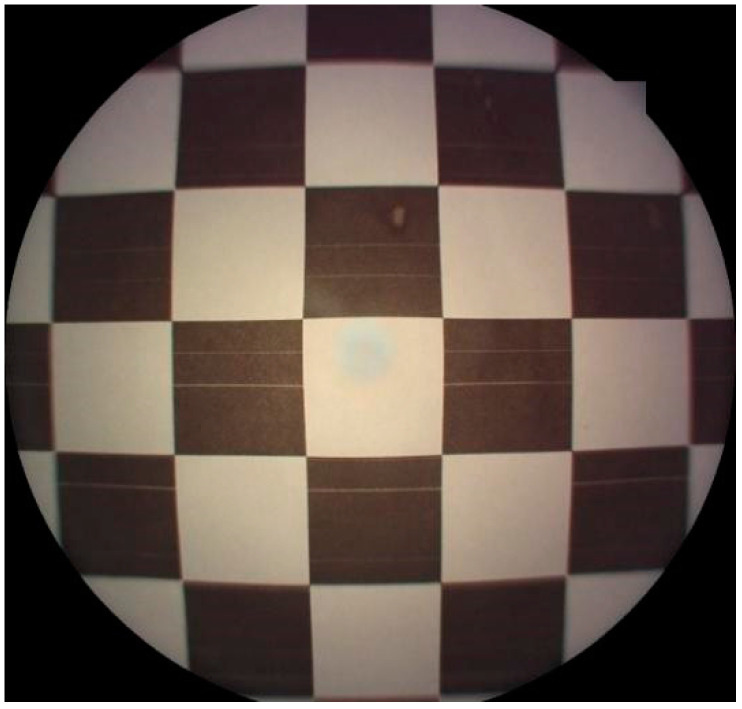
The image of a checkerboard captured by a fundus camera.

The pixel pitch is not constant in the raw fundus image, owing to the distortion of the camera lens, as shown in Figure 2. Generally, the pixel pitch at the edge of the fundus image is greater than that at the center of the image, and the distortion increases rapidly with distance from the center. In this sense, the average pixel pitch of the whole image is usually markedly greater than that at the center, while the pixel pitch around the optic disc is much closer to that at the center.

The ratio of the diameter of the ROI and that of the optic disc in the pixel is

(5) $$r=\frac{l_{ROI}}{l_{disc}}=\frac{L_{ROI}P_{disc}}{L_{disc}\bar{P}}=\frac{\alpha \left(f_{1}+\Delta d\right)P_{disc}}{L_{disc}\bar{P}}=\frac{l_{ROI}P_{disc}}{L_{disc}}$$

where $l_{disc}$ is the length of optic disc diameter in pixel, and $P_{disc}$ is the average pixel pitch around the optic disc.

Here, $P_{disc}$ is proportional to $\frac{f_{1}+\Delta d}{f_{1}}$, otherwise known as $P_{disc}\propto \frac{f_{1}+\Delta d}{f_{1}}$, for the images in which the position of the disc in the image is approximately constant, and it is stable for emmetropic or slightly-ametropic eyes. Consequently, according to Formula (5), the value of r only varies with Δd and $L_{disc}$ for the macula-centered images captured by a specific camera. In addition, as the average values of Δd and $L_{disc}$ can be relatively stable, and the value of r will be approximately constant if it is computed from the average length of optic disc diameter and ROI diameter in a pixel.

Conversely, average pixel pitch around the optic disc can be estimated by Formula (6) if the ROI–disc ratio is known,

(6) $$P_{disc}=\frac{rL_{disc}}{l_{ROI}}$$

(2) The workflow to estimate the average pixel pitch

**Figure 3:**
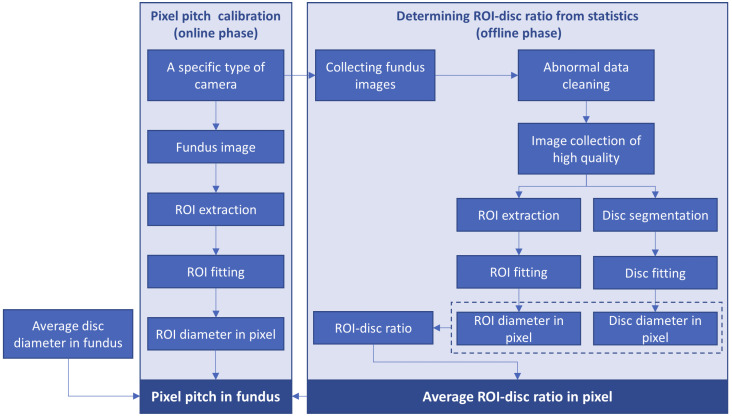
The workflow of estimating pixel pitch of fundus image.

In consequence, given the following constraints or assumptions, the average pixel pitch around an optic disc can be obtained by the workflow described in Figure 3.

(a) The average diameter of the optic disc is stable;
(b) the average axial ametropia of the eye is stable or can be ignored;
(c) the fundus images are captured with the optic disc approximately located at the same region;
(d) the FOV of the camera is constant;
(e) the distortion model of the camera lens is constant.

Constraint (c) is satisfied, as the images are captured with the macula at the center of the image, and assumptions (d) and (e) are met if the images are captured by the same camera. Assumptions (a) and (b) can be met according to the statistics data, which will be shown in the Results section.

According to Figure 3, the workflow can be divided into two phases, i.e., the offline statistical calibration phase and the online calibration phase.

**Figure 4:**
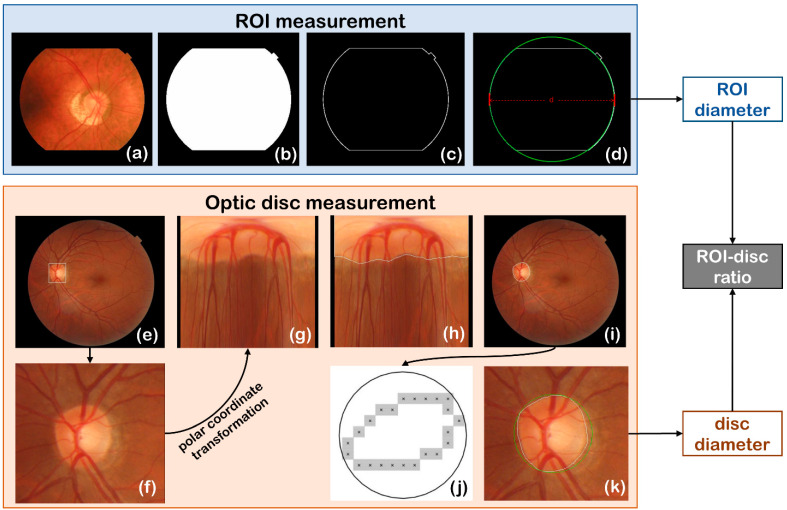
Automated ROI measurement and optic disc measurement. (**a**–**d**) are the steps of detecting ROI, (**e**) shows the optic disc position target detection box annotation, (**f**) is the detected region of optic disc, (**g**–**i**) are the steps of disc edge detection in polar coordinate system, and (**j**,**k**) show the circumscribed circle fitting of the optic disc.

In the offline phase (to find the ratio of the diameter of the ROI and that of the optic disc in a pixel): a large number of (usually thousands of) macula-centered fundus images captured by a specific type of fundus camera are collected, and the abnormal data, e.g., vague or low-contrast images and those of serious axial ametropia, are excluded. Then, the automated algorithms (as shown in Figure 4), which will be introduced later in Section 2.2.2 and Section 2.2.3, are applied to detect and measure the diameters of ROI and optic disc in the fundus image, and an average ROI–disc ratio in the pixel can be obtained by processing all the valid fundus images captured by the specific camera.

In the online phase (to estimate the pixel pitch of a fundus image): for a macula-centered fundus image, the ROI diameter in the pixel can be measured by the algorithm in Section 2.2.2, and the pixel pitch in fundus (near the optic disc) can be estimated according to Formula (6), given the ROI–disc ratio in pixel obtained in the offline phase.

The detailed methods in the workflow are introduced in Section 2.2.2, Section 2.2.3 and Section 2.2.4.

#### 2.2.2. Automated ROI Detection

**Figure 5:**
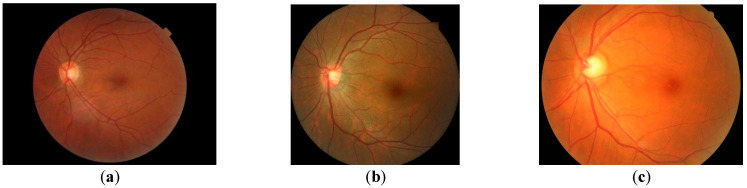
ROI of fundus image. (**a**–**c**) are examples of ROI regions that are smaller, roughly the same size as, and larger than the fundus image, respectively.

ROI is the valid area on the fundus image, that is, the area where fundus structures can be seen in the fundus image. It is generally located in the center of the image and is a circular area (Figure 5a). However, due to the difference of camera parameters, the shape of ROI may not be a complete circle in the fundus image, but it may be cropped to various extents (Figure 5b,c). Therefore, the ROI detection algorithm should be able to accurately identify the diameter of ROI when it is an incomplete circle.

The automatic extraction of ROI diameter in this study includes the following steps:

(1) First, we perform channel separation on the fundus image, select the red channel with the highest brightness as the extraction channel, and use 1/3 of the mean gray value of the red channel as the threshold to binarize the image.
(2) Then, areas of the bright connected-regions in the binary image are measured, and the connected-region with the largest area is the candidate of ROI (Figure 4b).
(3) The Canny operator is used to detect the edge of ROI (Figure 4c).
(4) Finally, the circular Hough transform is performed based on the edge of ROI, and the diameter of the circle with the most votes is the ROI diameter (Figure 4d).

#### 2.2.3. Automated Optic Disc Measurement

In this paper, the optic disc diameter is defined as the diameter of the minimum circumscribed circle fitting of the optic disc region. The acquisition of optic disc diameter mainly includes three steps: optic disc locating, edge detection, and minimum circumscribed circle fitting.

(1) Optic disc locating

In this study, we locate the disc based on SSD (Single Shot Detection), a deep learning object detection network model. Resnet50 is used as the backbone network of the model, six scales are used in the prediction layer, the image size of the input network model is 256 × 256, and the center point of the target detection box is used as the center point of the optic disc to locate the position of the optic disc.

The model training sample consists of 4100 fundus images and the corresponding optic disc position target detection box (as shown in Figure 4e), in which the target detection box is marked by the clinician.

(2) Edge detection of optic disc

Taking the center point of the optic disc obtained from the target detection network as the origin, polar coordinate transformation is performed on the fundus image (Figure 4g), Canny operator is applied to detect the optic disc edge under polar coordinates (Figure 4h), and the edge of the optic disc in the Cartesian coordinate system is finally obtained after inverse transform of the polar coordinate (Figure 4i).

(3) Minimum circumscribed circle fitting of optic disc

A minimum circumscribed circle algorithm is applied to fit the edge of optic disc to a circle (Figure 4j,k). In the set of pixels $\left\{P_{n}\left(x_{n},y_{n}\right)|n=1,2,\cdots ,N\right\}$ (N is the number of pixels on the edge of the optic disc) on the edge of the optic disc, two points, $P_{i^{*}}\left(x_{i^{*}},y_{i^{*}}\right)$ and $P_{j^{*}}\left(x_{j^{*}},y_{j^{*}}\right)$, with the largest Euclidean distance are found according to Formula (7):

(7) $$i^{*},j^{*}=\arg\max_{i,j}\sqrt{{\left(x_{i}-x_{j}\right)}^{2}+{\left(y_{i}-y_{j}\right)}^{2}}$$

The midpoint of the line between points $P_{i^{*}}$ and $P_{j^{*}}$ is the center of the optic disc, and the largest Euclidean distance $\bar{P_{i^{*}}P_{j^{*}}}$ is the diameter of the optic disc.

#### 2.2.4. Pixel Pitch Calibration

According to the average ROI–disc ratio obtained in the offline phase, there are two scenarios to estimate the pixel pitch around the optic disc based on Formula (6).

(1) Camera-specified calibration

For a specific fundus camera, the average ROI–disc ratio can be used to estimate the pixel pitch of other fundus images captured by the same type of camera. In this scenario, the FOV of the fundus camera can be arbitrary, but the average ROI–disc ratio can only be applied to a single type of camera.

(2) FOV-specified calibration

For the fundus cameras of the same FOV, the average ROI–disc ratio is supposed to be approximately constant despite small deviations caused by the differences in the lens distortion. To this end, the images of various fundus cameras of the same FOV can be used to calculate the average ROI–disc ratio for a specific FOV, which can be applied to estimate the pixel pitch around optic discs for difference cameras. In this scenario, the estimated pixel pitch is supposed to be less accurate than that obtained by camera-specified calibration since the average ROI–disc ratio used here might be slightly different from that of the images captured by the specific camera.

#### 2.2.5. Uncertainty Analysis

According to Formula (6), the uncertainty of $P_{disc}$ estimation can be calculated by Formula (8) following the theory of uncertainty propagation.

(8) $$U_{P_{disc}}=P_{disc}\sqrt{{\left(\frac{U_{r}}{r}\right)}^{2}+{\left(\frac{U_{L_{disc}}}{L_{disc}}\right)}^{2}+{\left(\frac{U_{l_{ROI}}}{l_{ROI}}\right)}^{2}}$$

where $U_{r}$, $U_{L_{disc}}$, and $U_{l_{ROI}}$ denote the uncertainty of r, $L_{disc}$, and $l_{ROI}$, respectively, and they can be obtained by calculating the standard deviation from the statistics data. Here, $L_{disc}$ can be derived from data of the existing studies according to Table 2, while $U_{r}$ and $U_{l_{ROI}}$ can be derived from the data used in this study, as described in Section 2.1.

Note that the ROI–disc ratio might vary by fundus camera due to the difference of lens distortion. If the camera-specified ROI–disc ratio is applied to estimate $P_{disc}$, $U_{r}$ can be calculated from the statistics data captured by the specific fundus camera; if a general ROI–disc ratio for different cameras of constant FOV is applied, however, $U_{r}$ needs to be derived from the statistics data captured by all the cameras.

### 2.3. Validation

#### 2.3.1. Validation of ROI and Optic Disc Detection

(1) Validation of ROI detection

The method described in Section 2.2.2 is used to identify the ROI of test sample pairs, and then the Accuracy (Acc), Sensitivity, Specificity, and Intersection over Union (IoU)—all of which are derived from confusion matrix—were used to evaluate and verify the detection results. For a specific type of camera, the ROI should be stable, so we use the statistical methods to verify the stability of the ROI diameter extraction method. The method in Section 2.2.3 is used to obtain the ROI diameter of the test sample, and then, the mean (*E*), the standard deviation, (*σ*) and the coefficient of variation (*V*), respectively, are calculated according to Formulas (9)–(11). Note that the coefficient of variation can eliminate the influence of data dimension and scale and reflect the divergence of the extracted ROI diameter. The smaller the coefficient of variation, the better the stability.

(9) $$E=\frac{1}{n}\sum_{i=1}^{n}x_{i}$$

(10) $$\sigma =\sqrt{\frac{1}{n}\sum_{i=1}^{n}{\left(x_{i}-E\right)}^{2}}$$

(11) $$V=\frac{\sigma }{E}$$

where n is the number of samples, and $x_{i}$ denotes the $i_{th}$ measurement.

(2) Validation of optic disc detection

The method in Section 2.2.3 is used to detect the optic disc from the test sample, and then, the Accuracy (Acc), Sensitivity, Specificity, and Intersection over Union (IoU) were used to evaluate and verify the detection results.

#### 2.3.2. Validation of Diameter Ratio of ROI and Optic Disc

Based on the auto-measured ROI diameters and optic disc diameters in the pixel, the distribution of the ROI–disc ratio can be obtained for each fundus camera, i.e., Canon CR2, Topcon NW400, Zeiss Visucam 200, and Newvision RetiCam 3100, as well as for all the cameras of 45° FOV. In addition, the mean value and standard deviation of the ROI–disc ratio are also calculated both cameras-wise and FOV-wise. The statistics results will be used to evaluate whether the diameter ratio of ROI and the optic disc is stable enough, to estimate the pixel pitches around optic disc, as well as to estimate the uncertainties for each camera.

#### 2.3.3. Validation of Distribution of Diopter

As the ROI-disc ratio and pixel pitch is related to the axial length or diopter of the eye, the average axial ametropia of the eye needs to be stable or negligible to support the proposed approach. Consequently, the database of Shanghai, as described in Section 2.1, is used to check whether or how the axial ametropia should be handled when the proposed approach is applied to estimate the pixel pitch.

#### 2.3.4. Reference Data Based on ISO 10940:2009

**Figure 6:**
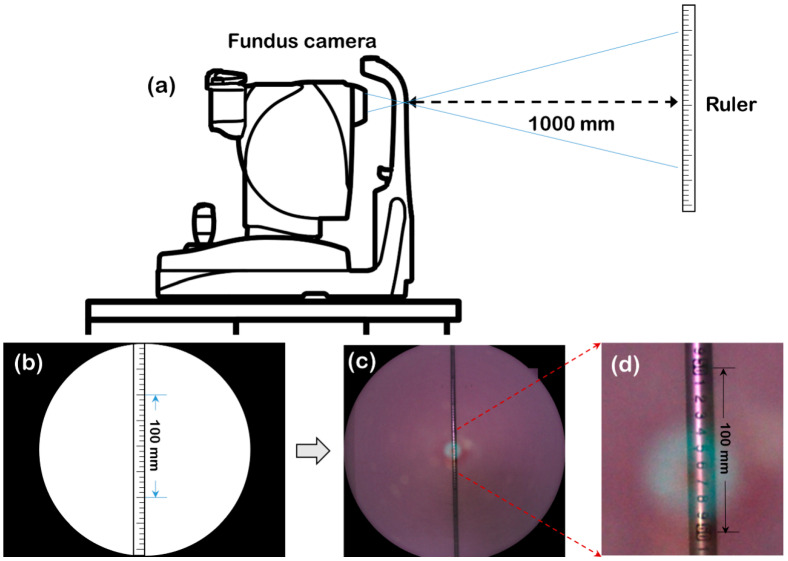
Diagram of pixel pitch measurement according to ISO 10940:2009. (**a**,**b**) are the schematic diagrams when shooting (place the ruler 1000 mm away from the pupil and ensure that the ruler scale in the center of the image can be seen clearly during shooting), (**c**) is the image acquired by actual measurement, and (**d**) is a partial enlarged view of (**c**).

According to ISO 10940:2009, the pixel pitch of the image is measured by taking an image of a 100 mm graduated target scale held on a screen 1000 mm away from the entrance pupil of the fundus camera (as shown in Figure 6). The number of pixels (*N*) covering the length of 100 mm on the image is measured to calculate the pixel pitch (in microns) of the fundus image using Formula (12).

(12) P=1700/N

The value 1700 results from the assumption that the eye is emmetropic and has a focal length of 17 mm.

## 3. Results

### 3.1. Results of ROI and Optic Disc Detection

(1) Accuracy of optic disc and ROI detection

The 2000 manually labeled samples were used to evaluate and verify the optic disc detection method and ROI detection method, respectively. The average confusion matrices of segmentation are listed in Table 3. According to the confusion matrices of 2000 validation samples, the average Acc, Sensitivity, Specificity, and IoU are 0.998, 0.969, 0.999, and 0.939 for optic disc detection and 0.993, 0.992, 0.994, and 0.990 for ROI detection.

The ROI can be easily segmented because it has a regular shape and differs significantly from the image background. As can be seen, the results of the four indicators were all higher than 0.99 for ROI detection. On the other hand, the Sensitivity (0.969) and IoU (0.939) were relatively lower for optic disc detection. The reason is that, even though the number of pixels falsely detected as background is small, the small optic disc area, as a percentage of the total image area, causes a clear decline in Sensitivity and IoU.

(2) Stability of ROI diameter measurement

We used a total of 24,000 fundus images for ROI diameter statistical analysis: 6000 fundus images were taken by Topcon, 6000 by Canon, 6000 by Zeiss, and 6000 by Newvision. The mean value, standard deviation, and coefficient of variation of measured ROI diameter are shown in Table 4.

### 3.2. Statistical Results of Diameter Ratio of ROI and Optic Disc

**Figure 7:**
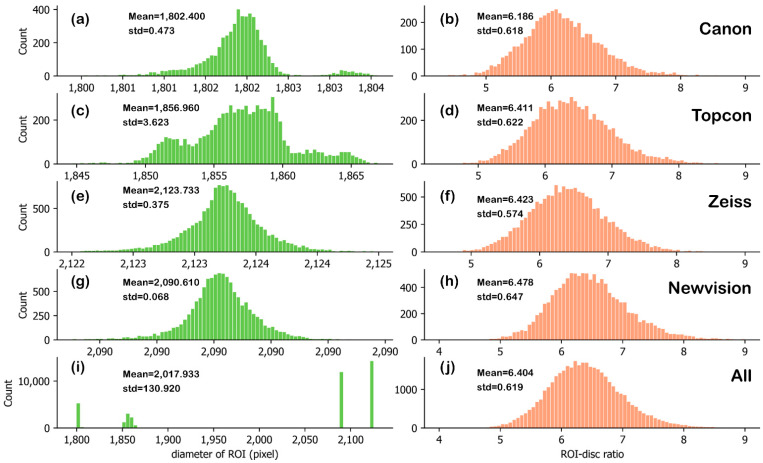
Statistics results of diameter of ROI and ROI–disc ratio for Canon (**a**,**b**), Topcon (**c**,**d**), Zeiss (**e**,**f**), Newvision (**g**,**h**), and all the cameras (**i**,**j**).

According to the auto-measured results, the distribution, mean value, and standard deviation (std) of the ROI diameters in the pixel and ROI–disc ratio for Canon, Topcon, Zeiss, Newvision, and four cameras together are shown in Figure 7.

One can find that both the diameter of ROI and the ROI–disc ratio are stable. Particularly, the ROI–disc ratio is normally distributed for each type of camera, with small standard deviations around 0.6. The mean value of the ROI–disc ratio of the Canon camera is a bit smaller than those of Tocon, Zeiss, and Newvision cameras, and it might result from the diverse lens distortions of the cameras. In this sense, for a specific camera, the average ROI–disc ratio can be applied to estimate the average diameter of the optic disc in the pixel, based on the diameter of auto-detected ROI.

In addition, although the ROI diameter varies for different cameras, the ROI–disc ratio is also normally distributed for the images captured by all the four types of fundus cameras, as shown in Figure 7j, and the standard deviation is 0.619. Since the ROI–disc ratio is stable for all the cameras of 45° FOV, the mean value of the ROI–disc ratio can be used to estimate the pixel pitches for different cameras of 45° FOV.

### 3.3. Statistical Results of Crowd Diopter

**Figure 8:**
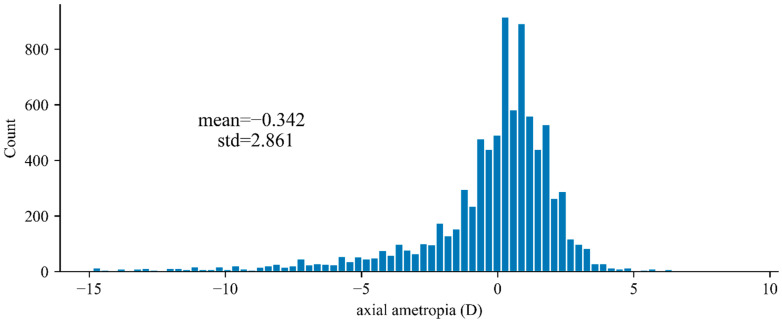
Distribution of axial ametropia of 4682 individuals (9364 eyes).

According to the database of Shanghai, the distribution of the axial ametropia of 9364 eyes of 4682 individuals is shown as Figure 8. It can be seen that most of the diopters of the eye are around zero, and the mean value and standard deviation of the diopter are −0.559 D and 3.522 D, respectively.

According to Formula (1), −0.342 D of Δd only results in less than 1% bias in fundus image magnification when $f_{1}$ is 17 mm. In consequence, the basic assumption (b) in Section 2.2 is true, and the average axial ametropia of the eye can be ignored when estimating the average pixel pitch using statistical method.

### 3.4. Results of Pixel Pitch Calibration

Based on ISO 10940:2009 method, the pixel pitch at the center of the image is measured several times for each fundus camera to reduce the random error. The final pixel pitches, obtained by averaging several measurements for each camera, are 6.897, 6.807, 5.693, and 6.050 for Canon, Topcon, Zeiss, and Newvision, respectively.

Based on the statistics of images captured by four types of fundus cameras, the pixel pitch calibration results are summarized in Table 5, including the ROI diameter in pixel ($l_{ROI}$), ROI–disc ratio (r), disc diameter in fundus ($L_{disc}$), and estimated pixel pitch around disc ($P_{disc}$) and their uncertainties ($U_{l_{ROI}}$, $U_{r}$, $U_{L_{disc}}$ and $U_{P_{disc}}$), as well as pixel pitches at the center of the image, measured based on ISO 10940:2009 ($P_{ISO}$), bias ($\Delta P_{disc}$), and relative bias ($\Delta {P^{\prime }}_{disc}$) of the estimated pixel pitch ($P_{disc}$), compared with $P_{ISO}$. $\Delta P_{disc}$ and $\Delta {P^{\prime }}_{disc}$ are calculated according to Formulas (13) and (14), respectively. The estimated pixel pitch and relative bias results are highlighted in bold.

(13) $$\Delta P_{disc}=P_{disc}-P_{ISO}$$

(14) $$\Delta {P^{\prime }}_{disc}=\frac{\Delta P_{disc}}{P_{ISO}}\times 100\%$$

According to Table 5, in both scenarios, the estimated pixel pitches are consistent with the ISO-based measurements taking the uncertainties, which make up around 10% of the estimation, into account. The actual biases of the estimation obtained by the proposed method are all less than 5% of the ISO-based measurements.

### 3.5. An Application of the Proposed Method

**Figure 9:**
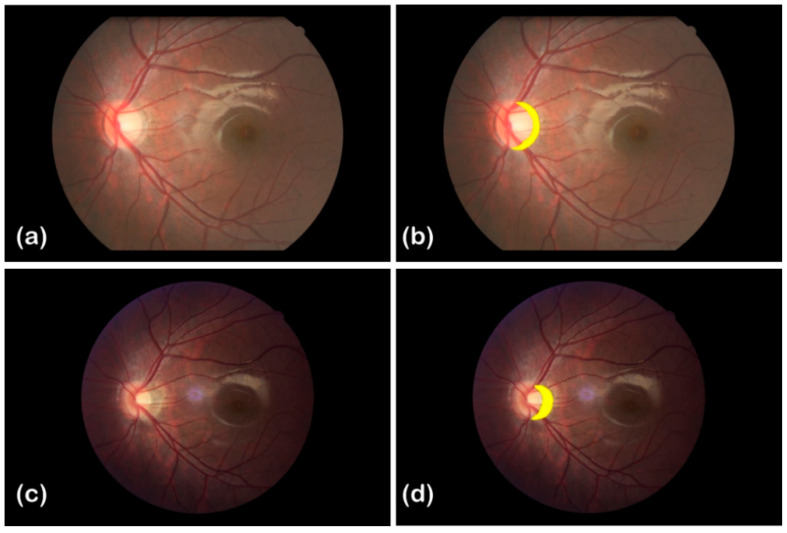
Fundus images of a myopic patient in 2012 and 2016. (**a**,**c**) are the fundus images taken by Cannon cameras of different models in 2012 and 2016, respectively. (**b**,**d**) are the labeled versions of (**a**,**c**), respectively, and show the regions of the atrophic arc highlighted in yellow. The areas of the atrophic arc regions are 17,586 px and 16,521 px in (**b**,**d**), respectively.

Here is an example of follow-up examination of a myopic patient in 2012 and 2016, as shown in Figure 9 and Table 6. The results of the left eye examination in 2012 were: SE (spherical equivalent refraction) = −2.50 D, AL (axial length) = 25.04 mm, and the clinical diagnosis was mild atrophy around the disc. Figure 9a shows the corresponding fundus image: the atrophic arc region is highlighted in yellow, and it covers 17586 px in the image. The results of the left eye examination in 2016 were: SE = −5.50 DS, AL = 27.47 mm, and the clinical diagnosis was moderate atrophy around the disc. The corresponding fundus image is shown as Figure 9b, and the total area of the atrophic arc region (highlighted in yellow) is 16,521 px.

According to the clinical diagnosis results of the two examinations, the degree of atrophy around the disc of the myopic patient was worsened. However, the fundus images showed that the atrophic arc region’s area, in pixels, had significantly decreased. It is a result of the two fundus images being taken by cameras with various pixel pitches. The pixel pitches were determined to be 5.99 μm and 7.36 μm, respectively, using our calibration method. After pixel pitch correction, the areas of the atrophic arc region were actually 0.631 mm^2^ in 2012 and 0.895 mm^2^ in 2016, and they were consistent with the clinical diagnosis.

This example demonstrates that it is impossible to compare the retinal structures directly between different fundus images without risking a disease diagnosis that is incorrect. On the other hand, the method suggested in this work can help the computer-based follow-up of disease progression by making different fundus images measurable and comparable.

## 4. Discussion

(1) The value of the method for quantitative analysis of fundus images and medicine

Since the fundus camera was successfully developed in 1925 [42], with the unremitting efforts of many researchers, the quality of image obtained by camera has been continuously improved, and it has also led to the increase in fundus camera manufacturers and camera types. The parameters of different camera types are often inconsistent, which makes the measurement of retinal structural parameters inconsistent in fundus images taken by different cameras, affecting the observation of clinical fundus features, especially the discovery of microscopic changes. In this paper, the ROI and disc size of fundus photos taken by different cameras are studied. It is found that the average diameter ratio of ROI and disc is 6.404 for cameras of 45° FOV, which was used as a reference to calibrate the pixel pitch of different types of fundus cameras. It solves the problem of inconsistent measurement of fundus structure caused by different camera parameters, which is conducive to the fine measurement of fundus structure, the establishment of data standards, and is of great significance for clinical diagnosis and treatment of ophthalmic diseases and early detection of systemic diseases.

In recent years, numerous studies have focused on the relationship between retinal vascular diameter, optic disc morphology, and diseases. Therefore, how to measure fundus structural parameters accurately and quickly is very important. However, the current mainstream software used to quantify the structural characteristics of the retina (e.g., SIVA) often requires extensive manual correction operations, which is not only time-consuming and laborious but also requires well-trained operators. Therefore, it is difficult to be applied in clinical practice, and it is difficult to carry out clinical research based on large data. At the same time, the measurement heterogeneity caused by manual operation has always been a sore point of research. This study not only achieves the pixel pitch calibration of different cameras, but it is also objective, which is of great significance to reduce the variability and measurement cost of retinal structural parameters in research. At present, we are not the only full-automatic method that can help measure retinal structural parameters. Future studies could compare our method with other software, such as SivA-DLS, QUARTZ, and ALTAIR [43,44,45].

With an accurate pixel pitch, we can quantify the lesions of retinal diseases (such as diabetic retinopathy and macular degeneration), monitor the progression of the disease, and monitor the effectiveness of the treatments. The patient’s systemic diseases can also be predicted by measuring the retinal structures, such as the size of the optic disc and the diameter of retinal veins and arteries [4,5,8,9]. For instance, the risk of developing cardiovascular and cerebrovascular diseases, such as hypertension, diabetes, stroke, myocardial infarction, etc., can be predicted using the calibration of the retinal blood vessels [4,5,8,9,46,47]. As a result, our findings are significant for disease prediction, diagnosis, and treatment.

(2) The uncertainty of the results

It should be noted that one of the bases of this study is the prior knowledge of average diameter of optic disc in fundus, i.e., 1.921 ± 0.026 mm, however, some studies [29,30,32] reported relatively smaller vertical diameters. Taking the uncertainty of the prior knowledge of average disc diameter into account, we find that, if the reported smallest vertical diameter, i.e., 1.82 mm [32], is used in the proposed method, the biases of the estimated pixel pitch obtained by the proposed method will be −6.2%, −7.8%, −3.6% and −7.8% for Canon, Topcon, Zeiss, Newvision, respectively, compared with the ISO-based measurements. In other words, the results of sensitivity analysis show that the possible bias resulting from the uncertainty of prior average disc diameter will not be greater than 10%.

Additionally, the proposed method is based on the statistics of big data, and the uncertainty of the estimation is mainly determined by the distribution of the samples. The uncertainties of the results are around 10%, according to the data used in this paper, and they can be reduced if more data are involved in the future. Particularly, the FOV-specified estimation only involved four fundus cameras, and the results will be strengthened if other cameras of 45° FOV are included.

Although the fundus images used in this work are macula-centered, the location of optic disc in the image can vary slightly and result in potential bias for the estimated pixel pitch. In addition, the proposed method is applied to estimate the pixel pitch around optic disc, which is generally a bit greater than that at the center of the image. The estimated pixel pitch can be more accurate if the lens distortion and the actual location of optic disc are modeled in the algorithm, and this will be taken into consideration in the future work.

(3) Applicable scenarios for the method

Firstly, the proposed method requires that the position of the optic disc in the image is approximately constant. In this work, the macula-centered images are used to make sure the location of optic disc is constant. However, as the optic disc is not near the edge of the macula-centered images, the pixel pitch around the optic disc will not deviate from the center pixel pitch too much, which can be seen from Table 5. In consequence, the result of this paper can also be applied to disc-centered fundus images without losing too much accuracy.

Secondly, the estimated pixel pitch based on the proposed method can only be applied to emmetropic or slightly ametropic eyes but not seriously ametropic eyes. The actual pixel pitch is related to the diopter of the eye—proportional to $\frac{f_{1}+\Delta d}{f_{1}}$, specifically. As a result, the error of the estimation of pixel pitch can be further compensated by Formula (15) if the diopter of the eye is available.

(15) $${P^{\prime }}_{disc}=P_{disc}\frac{f_{1}+\Delta d}{f_{1}}$$

where Δd is the positive or negative distance between the retina layer and the second focal plane of the eye, which can be calculated from Formula (2) if the axial ametropia of the eye is provided as D.

## 5. Conclusions

A generic and automated pixel pitch calibration approach for fundus camera is proposed in this paper. Without the knowledge of detailed specifics of the fundus camera, the average diameter of the optic disc in a pixel can be statistically obtained from a large number of images captured by a specific camera, and the pixel pitch can be estimated with the prior knowledge of average diameter of optic discs in fundus. In addition, for the images captured by different fundus cameras of the same FOV, the average diameter ratio of ROI and optic disc is supposed to be constant, and the pixel pitch can be quickly estimated according to the automatically measured diameter of ROI in pixel. In this paper, 40,600 fundus images with 45° FOV are analyzed, and ROI and optic discs of fundus images are automatically detected based on image processing technology. We can find that the average ROI–disc ratio is 6.404 ± 0.619, and based on this, the pixel pitch of fundus images taken by Canon CR2, Topcon NW400, Zeiss Visucam 200, and Newvision RetiCam 3100 are calculated respectively, the results are 6.825 ± 0.666 μm, 6.625 ± 0.647 μm, 5.793 ± 0.565 μm, and 5.884 ± 0.574 μm. Then, we compare the results with those measured according to ISO 10940:2009 method, and the results show that the deviation of pixel pitch measured by this method was less than 5%. It is proved that this method can directly obtain the pixel pitch around the disc area in fundus images with 45° FOV without knowing the type of fundus camera and its parameters. It can help to measure the real size of the optic disc accurately, and it also provides a reference for the accurate measurement of other fundus structures. As a result, when the camera type and its parameters are unknown, the fundus structures on the fundus image can be accurately measured with the pixel pitch obtained by this method. This method will be extremely important for the follow-up of disease progression and monitoring the efficacy of treatment methods with the help of fundus images.

## Figures and Tables

**Table 1:** Statistical sizes of human optic discs reported by the references.

ID	Ver. Dia./mm	Ver. Dia. Std./mm	Hor. Dia./mm	Hor. Dia. Std./mm	Notes
1	1.97	0.19	1.78	0.18	Color fundus images of 3918 normal subjects over 40 years old [28]
2	1.88	0.25	1.79	0.19	Color fundus images of 118 normal subjects [29]
3	1.88	0.19	1.71	0.20	Color fundus images of 475 subjects [30]
4	1.98	0.28	1.82	0.28	Color fundus images of 62 normal subjects [31]
5	1.82	0.16	1.59	0.17	OCT images of 110 young people [32]
6 *	1.84	——	1.84	——	Color fundus images of 4027 normal subjects, and only optic disc area is measured [33]
7 *	1.78	——	1.78	——	Clinical anatomy based on 14 people [34]
8 *	1.81	——	1.81	——	Color fundus images of 70 subjects [35]
9 *	1.76	——	1.76	——	Color fundus images of 5514 subjects over 55 years old [36]
10	1.96	0.20	1.84	0.20	Color fundus images of 122 subjects [37]
11 *	1.70	——	1.70	——	Color fundus images of 2634 subjects [38]
12	1.97	0.29	1.79	0.27	Color slides of 88 unselected optic discs [39]

* Note: these studies only provided areas of optic disc, and the vertical and horizontal diameters are approximately calculated from the areas.

**Table 2:** Meta-analysis of vertical diameter of optic disc.

Study Name	Statistics for Each Study					Sample Size	Relative Weight	Std Residual
	Mean	Standard Error	Lower Limit	Upper Limit	*p*-Value			
[28]	1.97	0.003	1.964	1.976	0	3918	15.744	0.819
[29]	1.88	0.023	1.835	1.925	0	118	14.043	−0.633
[30]	1.88	0.009	1.863	1.897	0	475	15.503	−0.670
[31]	1.98	0.036	1.910	2.050	0	62	12.185	0.849
[32]	1.82	0.015	1.790	1.850	0	110	14.966	−1.624
[37]	1.96	0.018	1.925	1.995	0	122	14.657	0.626
[35]	1.97	0.031	1.909	2.031	0	88	12.902	0.729
Results using meta analysis *	**1.921**	**0.026**	**1.870**	**1.972**	**0**			

* Note: The results were calculated using a random-effects model.

**Table 3:** The average confusion matrices of optic disc and ROI detection (Unit: pixels).

		Predicted Class (Optic Disc)		Predicted Class (ROI)	
		Positive	Negative	Positive	Negative
Actual Class (optic disc)	Positive	77,114	2467	——	——
	Negative	2543	2,539,982	——	——
Actual Class (ROI)	Positive	——	——	1,945,933	15,693
	Negative	——	——	3963	656,517

**Table 4:** Statistics of ROI diameter of cameras.

Camera Type	Image Size	Mean	Standard Derivation	Coefficient of Variation
Canon	2976 × 1984	1802.390	0.513	2.85 × 10^−4^
Topcon	1956 × 1934	1856.985	3.639	1.96 × 10^−3^
Zeiss	2124 × 2056	2123.710	0.481	2.27 × 10^−4^
Newvision	2091 × 1992	2090.593	0.238	1.14 × 10^−4^

**Table 5:** Results of pixel pitch calibration in camera-specified scenario and FOV-specified scenario for four types of fundus cameras.

	Camera-Specified Scenario				FOV-Specified Scenario			
	Canon	Topcon	Zeiss	Newvision	Canon	Topcon	Zeiss	Newvision
*l_ROI_*	1802.4	1856.96	2123.717	2090.61	1802.4	1856.96	2123.717	2090.61
$U_{l_{ROI}}$	0.473	3.623	0.469	0.068	0.473	3.623	0.469	0.068
*r*	6.186	6.411	6.423	6.478	6.404	6.404	6.404	6.404
*U_r_*	0.618	0.622	0.574	0.647	0.619	0.619	0.619	0.619
*L_disc_*/mm	1.921	1.921	1.921	1.921	1.921	1.921	1.921	1.921
$U_{L_{disc}}$/mm	0.026	0.026	0.026	0.026	0.026	0.026	0.026	0.026
*P_disc_*/μm	**6.593**	**6.632**	**5.810**	**5.952**	**6.825**	**6.625**	**5.793**	**5.884**
$U_{P_{disc}}$/μm	0.665	0.650	0.525	0.600	0.666	0.647	0.565	0.574
*P_ISO_*/μm	6.897	6.807	5.693	6.050	6.897	6.807	5.693	6.050
∆*P_disc_*/μm	−0.304	−0.175	0.117	−0.097	−0.071	−0.182	0.100	−0.165
∆*P′_disc_*/%	**−4.402**	**−2.567**	**2.061**	**−1.609**	**−1.033**	**−2.673**	**1.759**	**−2.733**

**Table 6:** Results of follow-up examination of a myopic patient in 2012 and 2016.

Year	Area of Atrophic Arc Region/px	Area of Atrophic Arc Region/mm^2^	Clinical Diagnosis		
			Spherical Equivalent Refraction (SE)/DS	Axial Length (AL)/mm	The Degree of Atrophy around the Disc
2012	17,586	0.631	−2.50	25.04 mm	mild
2016	16,521 **↓**	0.895 ↑	−5.50 ↑	26.47 mm ↑	moderate

Note: The up arrow ↑ means the indicators raised, and the down arrow **↓** means the indicators declined.
